# Restricting Human Movement During the COVID-19 Pandemic: New Research Avenues in the Study of Mobility, Migration, and Citizenship

**DOI:** 10.1177/01979183221118907

**Published:** 2022-11-15

**Authors:** Lorenzo Piccoli, Jelena Dzankic, Didier Ruedin, Timothy Jacob-Owens

**Affiliations:** 10185 European University Institute, Florence, Italy; University of Neuchatel, Neuchatel, Switzerland; 10185 European University Institute, Florence, Italy; 27214 University of Neuchatel, Neuchatel, Switzerland; 10185 European University Institute, Florence, Italy

**Keywords:** mobility, COVID-19, migration

## Abstract

Every government in the world introduced restrictions to human mobility – that is, the movement of persons across and within state borders – in response to the COVID-19 pandemic. Such restrictions thus constituted a global phenomenon, but they were by no means globally uniform; rather, they varied significantly between and within states, as well as over time. This research note presents different data sources for studying the drivers and outcomes of mobility restrictions, highlighting specific ways in which the data can be used. We begin by surveying seven new databases capturing various aspects of the regulation of human movement during the COVID-19 pandemic. Drawing inspiration from research on previous pandemics, we then outline five possible research avenues prompted by these data. We suggest that explaining the causes and consequences of such restrictions, as well as the differences between them, can significantly advance research on the governance of mobility, migration, and citizenship.

## Introduction

Every government in the world introduced restrictions to human mobility – that is, the movement of persons across and within state borders – in response to the COVID-19 pandemic. Although such restrictions thus constituted a global phenomenon, they were by no means globally uniform; rather, they varied significantly between and within states, as well as over time. For instance, while the majority of countries closed their external borders to most travellers, a small minority – among them Ireland, Mexico, and the United Kingdom (UK) – opted to rely on less drastic travel restrictions. The border closures were themselves highly variable, with some lasting only a few months (e.g., in Brazil) and others the best part of a year (e.g., in South Africa). During the “closures,” many states continued to grant entry to certain categories of travellers, often including permanent residents, diplomats, and transport personnel, while others (e.g., Australia and Morocco) even barred entry to their own citizens. As the pandemic has worn on, states have increasingly moved away from border closures to travel restrictions based on medical documentation, such as negative COVID-19 tests and vaccine certificates. The nature, scope, and duration of “internal” mobility restrictions, such as lockdowns, curfews, and regional boundary closures, have likewise varied considerably across the world.

The global scale and variation of COVID-19 mobility restrictions open new avenues for social scientific inquiry. Early studies of the restrictions focused on their epidemiological effects (e.g., Chinazzi et al. 2020; Lee and Lee 2020; Koopmans 2021; Wells et al. 2020) and their impact on patterns of human movement (Guadagno 2020; Iacus et al. 2020; Martin and Bergmann 2021; Pepe et al. 2020; Santamaria et al. 2020). By contrast, this research note highlights the significance of global variation in the restrictions as such and, in particular, its implications for the “global mobility regime” – that is, the legal and policy frameworks producing unequal opportunities to travel across and within state borders (Glick Schiller and Salazar 2013; Koslowski 2011). From this perspective, the restrictions not only impact on the movement of persons across and within state borders (mobility) but also the movement of persons away from their usual place of residence (migration) and the legal relationship between persons and states as recognised under international law (citizenship).

Research conducted in the context of previous pandemics generated five key insights concerning mobility restrictions. First, during pandemics, countries limit human movement not only in response to changing global epidemiology but also according to other criteria, including diplomatic and economic considerations (Abeysinghe 2016; Amon and Todrys 2008; Markel and Stern 2002; Siewe, Lenhart, and Yakubu 2020). Second, during pandemics, different communities tend to use broadly similar measures to curtail human mobility (Bier 2020; Clemens and Ginn 2020; Tognotti 2013). Third, travel restrictions introduced during pandemics cause significant economic disruption to affected communities (Cetron and Landwirth 2005; Colizza et al. 2007; Epstein et al. 2007; Schabas 2004). Fourth, restrictions have often been accompanied by exceptions for specific groups based on their legal or professional status (Snowden 2019; Vanderslott and Marks 2021). Finally, restrictions sometimes outlasted the emergency they were meant to contain, creating new categories of desirable and undesirable travellers (Rushton 2012).

These earlier findings suggest that conducting research on the COVID-19 travel restrictions can significantly advance our understanding of mobility, migration, and citizenship governance during and after the pandemic. This research note is intended as a catalyst for such efforts. In the next section, we present a survey of seven new datasets capturing various aspects of the regulation of human movement during the COVID-19 pandemic. We then outline five possible research avenues prompted by these data, drawing inspiration from the earlier insights outlined above.

## Available Data

Data capturing the duration, scope, timing, and target population of mobility restrictions introduced during the COVID-19 pandemic are already available. Here, we limit our survey to longitudinal datasets that track the regulation of human movement during the pandemic and enable the comparison of mobility restrictions across different countries. We also exclude datasets that do not comply with the FAIR data principles (Wilkinson et al. 2016). On this basis, we identified six relevant datasets, with a seventh in the making.^1^ Although all identified datasets focus on governmental responses to the crisis, they vary substantially in terms of the measures/policies and governance levels they capture. Consequently, they have different potential and limitations for addressing different research questions. We summarise the main characteristics of each dataset in Table 1, before discussing their strengths and weaknesses in greater detail.

**Table 1.** Available Data on the Regulation of Human Movement During COVID-19.

Dataset	Geographical Coverage	Temporal Coverage	Updates	Type of Data	Movement-Related Indicators	Data Sources
CoronaNet project	201 countries	February 2020 – ongoing	Weekly	Intervention	Lockdowns, internal border restrictions, and external border restrictions	Government websites, newspapers
COVID Border Accountability Project (COBAP)	246 countries and associated island territories	February 2020 – December 2020	—	Intervention	International travel restrictions (complete closure vs partial closure)	Government websites, newspapers
COVID-19 Mobility Tracking Database	184 countries, territories, or areas	March 2020 – ongoing	Weekly	Country-day	Type and targets of international travel restrictions	International Air Transport Association (IATA)
International Travel Restrictions in Response to Covid-19 Dataset	212 countries	February 2020 – June 2021	Biannually	Country-day	Type and targets of international travel restrictions and related exemptions	Government websites, newspapers, International Organization for Migration (IOM) reports
Mobility and Border Control in Response to Covid-19 Dataset	32 countries: European Union (EU), European Free Trade Association (EFTA), and the United Kingdom (UK)	February – June 2020	—	Intervention	Lockdowns and international travel restrictions	Government websites, newspapers
Lex-Atlas: Covid-19 (LAC19)	59 countries	February 2020 – ongoing	Monthly	Intervention	National legal responses to COVID-19	Government websites
Oxford COVID-19 Government Response Tracker (OxCGRT)	185 countries	January 2020 – ongoing	Weekly	Country-day	Containment and closure measures impacting human mobility	Government websites

*Source:* Own elaboration.

### CoronaNet Project

The CoronaNet project (Cheng et al. 2020) codes policy announcements published on government websites across 201 countries starting from February 2020 (the dataset is still ongoing). The database covers 18 broad policy interventions. Three of these interventions are directly related to the governance of mobility: quarantines, border closures, and curfews. Two other interventions indirectly limit human movement: the closure of non-essential businesses and schools, and restrictions on mass gatherings. The dataset includes over 110,000 policy interventions. The scope for analysis of international travel restrictions is limited because the dataset only covers the closure of borders and does not record other restrictions and related exceptions. Unlike other datasets, however, CoronaNet captures policies regarding restrictions between municipal and regional borders, consistently recording the level of government (local, regional, national) that initiated a given policy measure. This dataset can be used for large-N comparisons aimed at identifying the drivers of different restrictions over time and across space.

### COVID Border Accountability Project (COBAP)

The COVID Border Accountability Project (COBAP) (Shiraef et al. 2021) records international travel restrictions in 246 states and territories worldwide. The database includes detailed information on international mobility restrictions: what type of closures was introduced, which exceptions were made, which countries were banned, and which borders were closed. The dataset includes over 1,000 policies collected from government websites and newspapers. Unlike other datasets, COBAP makes all original source links available. It also provides an aggregated indicator of “complete closure” and “partial closure” that simplifies comparative analysis. Although preliminary data are available for 2021, the authors only ensure consistency for the period February–December 2020. This dataset can be used for tracking the diffusion of international travel restrictions in different regions of the world.

### COVID-19 Mobility Tracking Database

The COVID-19 Mobility Tracking Database is produced by the International Organization for Migration (IOM 2021). It covers 184 countries, territories, or areas and is generated using data from the International Air Transport Association (IATA), with weekly updates starting from February 2020 (the dataset is still ongoing). The dataset includes information on the types and targets of international travel restrictions. Restrictions are aggregated into six major groupings: Route Restrictions (RC) that apply to all travellers arriving from or transiting through a specific country, territory, or area, regardless of their nationality; Nationality Restriction (RN) that apply to travellers with specific nationalities; Visa Changes (VC) that entail changes in visa policy, such as suspension of visa on arrival, visa invalidations and other emerging measures; Document Changes (DC) that include changes to mobility agreements impacting upon the documentation (passport or ID) required for passengers or nationals arriving from a specific country; Other Limitations (OL) that apply to limitations that do not fall under the previous categories and other emerging measures; Conditions for Authorised Entry (CAE) that apply to medical/health related or other measures that are necessary to fulfil to enter a country, territory or area. These aggregations facilitate a comparative analysis of the different types of international travel restrictions that were introduced during the pandemic. The data are available only upon request.

### International Travel Restrictions in Response to Covid-19 Dataset

The “International Travel Restrictions in Response to Covid-19 Dataset” (Piccoli et al. 2020a, 2021) covers international travel restrictions between February 2020 and June 2021: entry bans, requirements of negative COVID-19 test results/vaccination, medical screening, quarantines, self-isolation, and suspension of visa-free agreements. The dataset covers the targets of and exemptions from each restriction in 212 countries and territories. Data are collected using the Human Mobility Impacts reports of the International Organization for Migration (IOM), government websites, and online media. The dataset includes over 130,000 separate episodes, allowing detailed cross-national studies. At the same time, the granularity of the restrictions and the lack of aggregate indices make it difficult to straightforwardly produce comparative analyses. This dataset can be used for comparing which groups of travellers were most frequently targeted by international travel restrictions in different regions of the world.

### Mobility and Border Control in Response to Covid-19 Dataset

The “Mobility and Border Control in Response to Covid-19 Dataset" (Piccoli et al. 2020b; Hoffmeyer-Zlotnik and Rausis 2021) covers the member states of the European Union, European Free Trade Association (EFTA), and the United Kingdom between February and June 2020. It tracks policies that restrict international mobility (closure of the borders, suspension of flights, and mandatory quarantine) and internal mobility (with a focus on the closure of non-essential businesses, confinement orders, and curfews). The dataset includes 375 episodes coded from government websites and newspaper agencies. Providing a standardised measure of closure for international and domestic mobility, this dataset can be used to compare the evolution of different restrictions across space. However, it covers a limited time span and is largely restricted to Western European countries. Hence, this dataset can be used most effectively for small-N studies on the evolution of travel restrictions in the early phases of the pandemic.

### Lex-Atlas: Covid-19 (LAC19)

The Lex-Atlas: Covid-19 (LAC19) (King and Motta Ferraz 2021) systematises quantifiable data concerning government responses to the pandemic, including travel restrictions, in 51 countries starting from February 2020 (the project is still ongoing). The LAC19 database captures further legal data, such as the types of law-making activity and emergency powers used in response to the pandemic. The database allows for comparison using socioeconomic and political variables, such as government regime type, income, and type of legal system, albeit across a limited range of countries. The database does not, however, include indicators that would allow direct comparison across countries.

### Oxford COVID-19 Government Response Tracker (OxCGRT)

The Oxford COVID-19 Government Response Tracker (OxCGRT) (Hale et al. 2020a; Hale et al. 2020b) provides data on measures introduced in 185 countries, including but not limited to mobility restrictions. The dataset was started in February 2020 and is still being updated at the time of writing. The data are gathered from governments’ websites and are organised around 23 policy indicators, eight of which concern “containment and closure” measures impacting upon human mobility: school closures, workplace closures, cancellation of public events, restrictions on gathering size, closure of public transport, stay-at-home requirements, restrictions on internal movement, and restrictions on international travel. Unlike other datasets, policies are recorded on a scale to reflect the extent of government action, and scores are aggregated into a suite of policy indices for each national government. This makes the dataset best suited for large-N comparisons aimed at understanding the type and duration of restrictions adopted by different countries. This dataset primarily not only covers responses at the national level but also includes regional/state governments in Brazil, the United Kingdom, and the United States, as well as city-level authorities in Brazil. However, the indicators on international travel restrictions aggregate a variety of measures and therefore do not differentiate, for example, between border closures, quarantines, and medical testing at national borders.

## Research Avenues

The datasets presented above offer a rich resource for social scientific inquiry, broadly defined. In this section, we outline five possible avenues for future research on the governance of mobility, migration, and citizenship, building on insights generated by earlier scholarship on previous pandemics.

### Research Avenue 1: The Drivers of COVID-19 Mobility Restrictions

The global variation in COVID-19 mobility restrictions raises the question of why different governments made different policy choices in response to the pandemic. The drivers of these choices could include medical and epidemiological concerns (e.g., number of cases in the target countries), party ideology (e.g., liberal governments may be more reluctant to restrict mobility), transnational alliances (e.g., formal trade and mobility agreements between countries may limit the introduction of reciprocal travel bans), policy learning (e.g., experience with previous epidemics such as SARS, MERS or Ebola), structure of government (e.g., federal countries may be slower to introduce restrictions), and economic policy (e.g., reliance on migrant workers may push states not to restrict labour-based mobility). Furthermore, we expect domestic and international mobility rules to be driven by different sets of expectations and policy dynamics.

**Figure 1.**
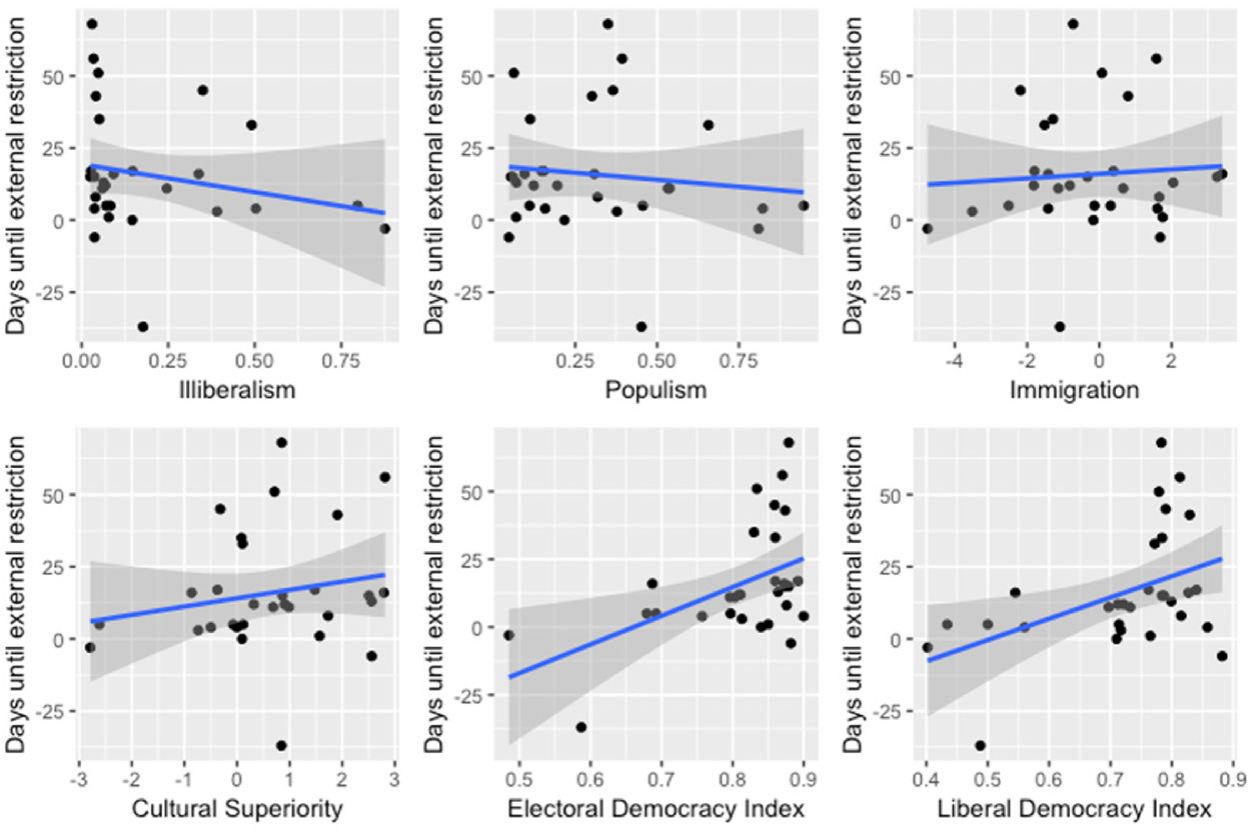
Timing of the First International Mobility Restriction Introduced, Correlated with Party Ideology and Level of Democracy (EU and EFTA countries).

*Source:* Own Elaboration Based on Cheng et al. (2020); Coppedge et al. (2021); Hoffmeyer-Zlotnik and Rausis (2021); Lührmann et al. (2020); Piccoli et al. (2020b).

To illustrate this avenue of research, we correlate the timing of states’ first international mobility restrictions (drawn from Cheng et al. 2020; Piccoli et al. 2020b) with two V-Dem datasets (Coppedge et al. 2021; Lührmann et al. 2020) to explore whether this timing is influenced by the “party ideology” of the governing party or the “level of democracy” in the state. This preliminary analysis suggests that party ideology does not have a significant effect on the timing of international mobility restrictions. By contrast, the level of democracy appears to be a relevant factor: The higher the level of democracy, the slower the introduction of restrictions (Figure 1).

### Research Avenue 2: Patterns of Policy Convergence and Divergence

**Figure 2.**
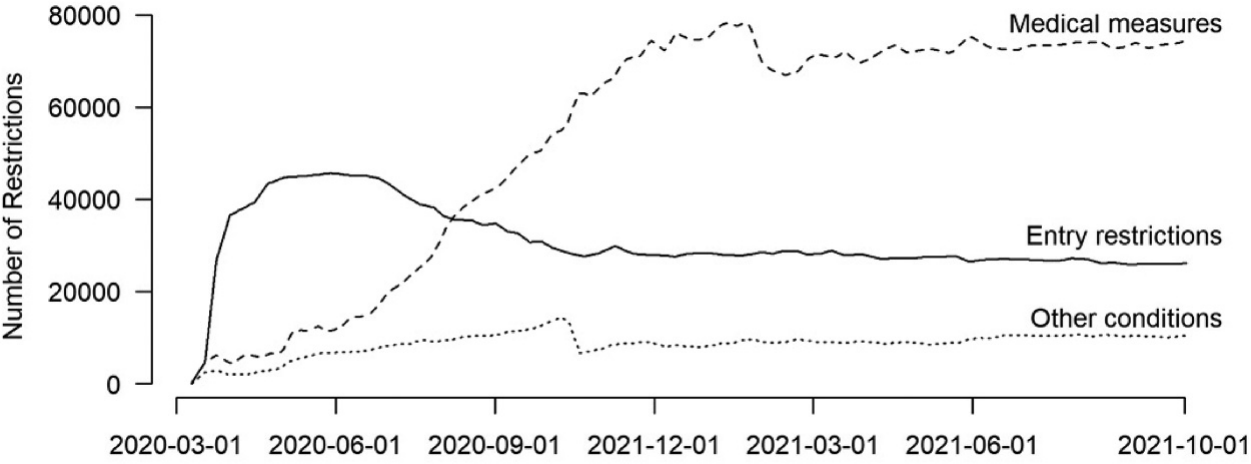
Evolution of International Travel Restrictions by Type Between March 2020 and October 2021.

*Source:* Own Elaboration Based on IOM (2021).

Comparative political scientists can use all seven datasets to reveal patterns of policy convergence and divergence and to explore international policy diffusion over time. For example, in June 2020, most states restricted entry but only few deployed public health measures as conditions for border crossing (swab, screening). By June 2021, the number of travel bans decreased, while the number of public health measures regulating entry significantly increased. Figure 2 illustrates this dynamic drawing on data from IOM (2021).

### Research Avenue 3: The Legality of Mobility Restrictions

The scope and duration of COVID-19 mobility restrictions raise questions regarding their compatibility with pre-existing legal norms permitting human movement. The data discussed in the previous section allow lawyers to track the emerging state practice regarding international mobility restrictions and assess this in light of state obligations under international human rights law and international refugee law (Scheinin and Molbæk-Steensig 2021; Hathaway et al. 2021). Article 12(4) of the International Covenant on Civil and Political Rights, for example, provides that “no one shall be arbitrarily deprived of the right to enter [their] own country.” As noted above, however, countries including Australia and Morocco have prevented the entry of their own citizens, thus potentially acting in breach of their international human rights obligations (Hicks 2021). Legal scholarship might also draw specifically on the data collected by King and Motta Ferraz (2021) to assess the constitutionality of internal mobility restrictions – that is, their compatibility with domestic constitutional norms limiting the reach of government powers and protecting fundamental rights, such as freedoms of association and assembly (Serna de la Garza 2020; Thompson and Ip 2020).

### Research Avenue 4: Continuity and Change in Global Migration Policy

**Figure 3.**
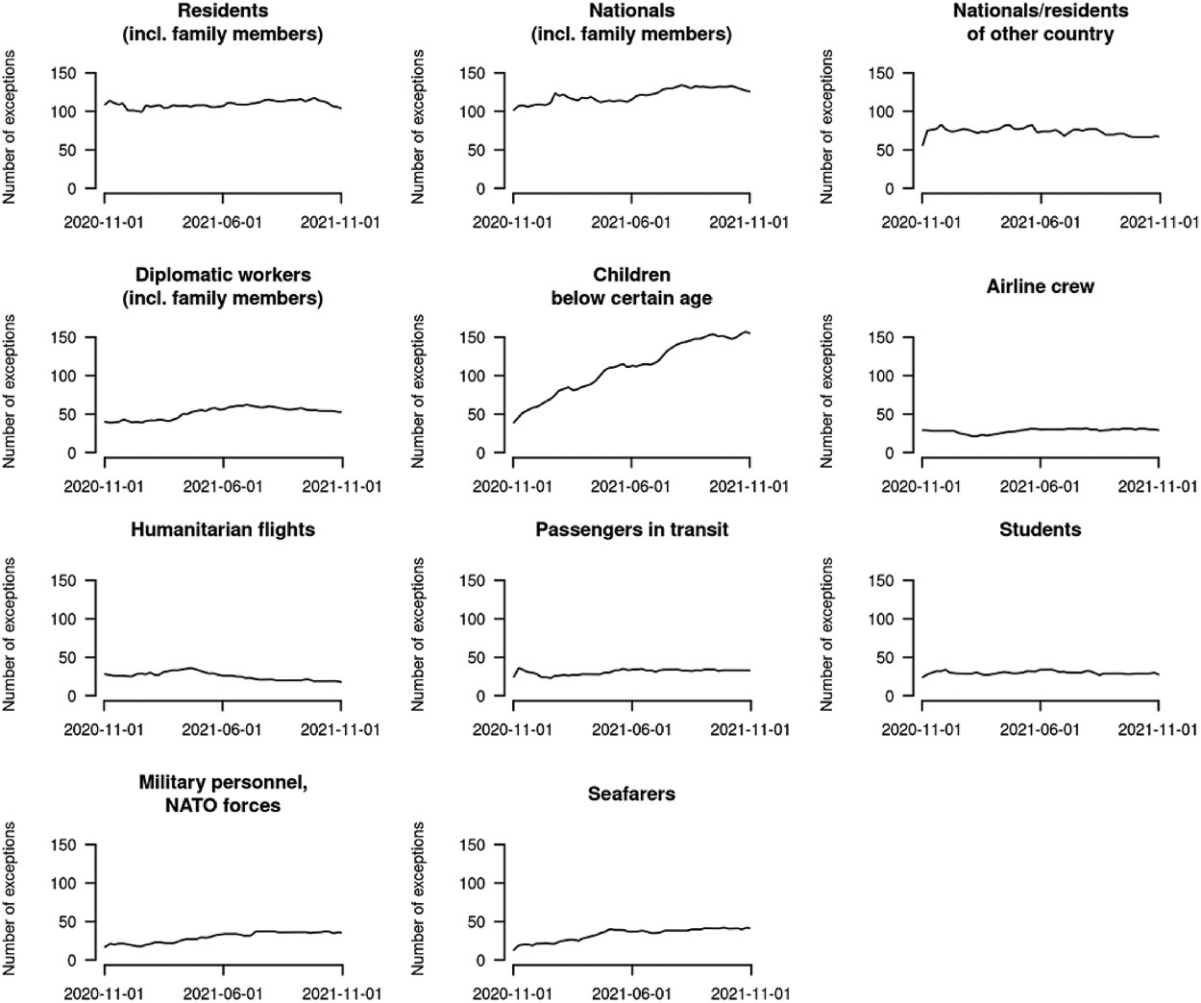
Evolution of the Exceptions to International Travel Restrictions Between November 2020 and November 2021.

*Source:* Own Elaboration Based on IOM (2021).

COVID-19 mobility restrictions did not target all individuals uniformly but rather depending on their country of origin and legal status (citizen, temporary resident, asylum seeker, and so on). In this way, the restrictions exemplified a broader trend in contemporary migration policies, which operate as a selection mechanism based on similar characteristics (Beine et al. 2016; De Haas et al. 2019). The datasets by IOM (2021) and Piccoli et al. (2020a, 2021) allow scholars to identify what type of mobility was still permitted during the pandemic – through analysis of the targets of and exceptions to COVID-19 mobility restrictions – and thus to explore the extent to which the restrictions represent instances of continuity or change within global migration policy. For example, an assessment of which migrant workers were exempt from international border closures – such as medical staff, transport personnel, or agricultural workers – could explore trends in the understanding of ‘essential’ labour migration during the pandemic (Anderson, Poeschel and Ruhs 2021; Fasani and Mazza 2020; Gelatt 2020). Figure 3 below shows the evolution over time in the number of countries granting exceptions for different categories of travellers.

### Research Avenue 5: Citizenship and International Mobility Rights

**Figure 4.**
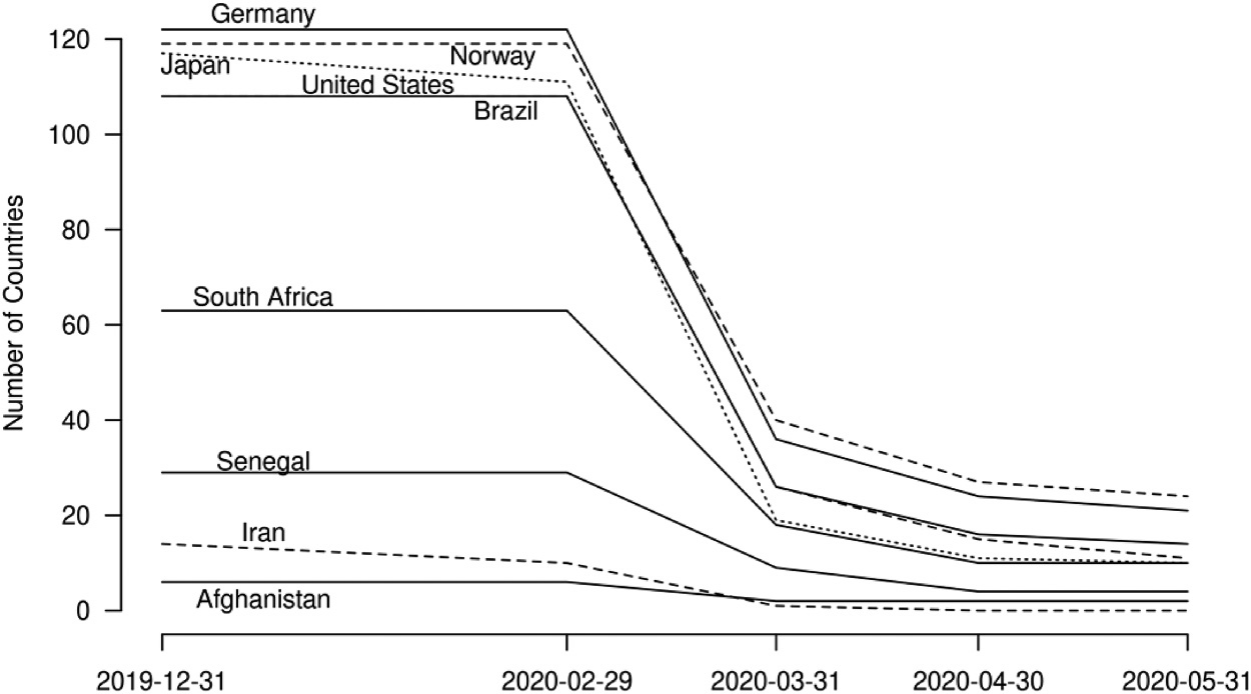
Relative Decline in the Number of Countries That Selected Passport-Holders Could Access without a Visa.

*Source:* Own Elaboration Based on Recchi et al. (2020) and Piccoli et al. (2020a).

Since before the pandemic, the right to cross state borders has been closely tied to a person's citizenship, with much greater international mobility rights traditionally accorded to citizens of states in the Global North (Czaika and De Haas 2017, Mau et al. 2015, Milanovic 2016, Recchi et al. 2020). COVID-19 mobility restrictions raise questions regarding the continued importance of citizenship for international mobility rights during the pandemic; for instance, whether citizens were still permitted to return to their country of origin and whether the citizenship of Global North states continued to guarantee more far-reaching international mobility rights. By way of example, Figure 4 below combines data on pre-pandemic visa-free travel (Recchi et al. 2020) with data on COVID-19 international mobility restrictions (Piccoli et al. 2020b). The results indicate that international travel restrictions had a greater effect on passport-holders from the Global North, because their pre-pandemic mobility rights were far more extensive than those of passport-holders from the Global South. In this way, COVID-19 mobility restrictions had an ‘equalising’ effect on citizenship-based international mobility rights. It remains to be seen how this trend will evolve as the pandemic recedes.

## Conclusion

In this research note, we have highlighted how newly available data on COVID-19 mobility restrictions can be used to explore the drivers of relevant policy choices, cross-border policy divergence and convergence, the legality of the restrictions, continuity and change in global migration policy, and the intersection between citizenship and shifting international mobility rights. Taken together, these five research avenues can serve to advance our understanding of the effects of a public health emergency on the “global mobility regime” (Glick Schiller and Salazar 2013; Koslowski 2011). The COVID-19 pandemic offers an unprecedented opportunity to examine this issue, given both the global scope and variation of the restrictions imposed and the wealth of available data concerning them. The pandemic may also constitute a key turning point in the future development of the global mobility regime. Our ability to understand its current effects may thus have implications for scholarly work for years to come.

## Notes

1 All references to “ongoing” and numbers are as of June 1, 2022.
